# Impact of Intracystic Hemorrhage on Therapeutic Outcomes in Macro/Mixed Cystic Lymphatic Malformation: A Retrospective Cohort Study

**DOI:** 10.3390/children12070935

**Published:** 2025-07-16

**Authors:** Tao Han, Daolin Ye, Jie Cui, Songming Huang, Weimin Shen

**Affiliations:** 1 Department of Burns and Plastic Surgery, Children’s Hospital of Nanjing Medical University, Nanjing 210008, China; dr.hantao@njmu.edu.cn (T.H.); yedaolin800@126.com (D.Y.); cuijunmeng@163.com (J.C.); 2 Department of Nephrology, Children’s Hospital of Nanjing Medical University, Nanjing 210008, China

**Keywords:** intracystic hemorrhage, cystic lymphatic malformation, indocyanine green, therapeutic outcomes, children

## Abstract

**Objectives**: This research aims to examine the impact of intracystic hemorrhage (ICH) on therapeutic outcomes in children with macro or mixed cystic lymphatic malformation (cLM). **Methods**: This retrospective study included macro/mixed cLM cases with or without ICH who underwent treatment between January 2019 and June 2024. All patients were diagnosed using preoperative imaging findings and intraoperative indocyanine green (ICG) lymphography. The baseline data of enrolled cases were retrospectively collected. The clinical characteristics were documented, including gender, age, histological typing, location, maximum diameter, and intracystic condition. Patients with or without ICH were divided into two groups. The dependent variables for predicting an excellent outcome were analyzed using multivariable logistic regression models after adjusting for potential factors using a univariable regression model. Postoperative variables, including duration of negative drainage, local infection, scar hyperplasia, and follow-up, were compared between the two groups. **Results**: A total of 83 cLM patients were included (ICH group: *n* = 36 and without ICH group: *n* = 47). A complete absence of afferent lymphatic vessels was demonstrated using intraoperative ICG lymphography, suggesting the isolated nature of ICH cases. ICH (*p* = 031; OR, 2.560; 95% CI, 1.089–6.020) was identified as the main predictor, and younger patients (*p* = 035; OR, 0.415; 95% CI, 0.183–0.940) had a lower potential for excellent outcomes. For the postoperative variables, the ICH group exhibited a shorter duration of negative drainage than the without ICH group (*p* < 0.001), while no significant differences were found regarding local infection (*p* = 0.693) and scar hypertrophy (*p* = 0.648). **Conclusions**: Although characterized by aggressive progression and compressive symptoms, ICH emerges as an independent favorable prognostic predictor in macro/mixed cLM management, potentially attributable to its isolated nature.

## 1. Introduction

Lymphatic malformation (LM) is a congenital, low-flow vascular malformation resulting from aberrant embryonic development, which leads to structural defects in the lymphatic vessels. It originates from lymphatic endothelial cells (LECs), and is characterized by dysregulated LEC proliferation and cystic dilation of lymphatic vessels. The most common type of LM is cystic lymphatic malformation (cLM), which is a congenital, low-flow vascular anomaly in children, typically occurring in regions rich in lymphoid tissue. Approximately 70% of cases arise in the head and neck, while 25% involve the trunk and extremities, and 5% affect the thoracoabdominal viscera [1]. Most affected infants present with localized symptoms at birth, including subcutaneous cystic masses with or without cutaneous discoloration [2]. Cervical cLM may lead to compression of the esophagus or trachea, resulting in dysphagia or respiratory distress. Craniofacial cLM is often associated with abnormal overgrowth of the maxilla or mandible, causing malocclusion, facial disfigurement, and psychological distress [3,4,5]. Periorbital cLM may manifest with exophthalmos, impaired ocular motility, and ptosis [6,7].

These cLM lesions occur as solitary lesions of variable size and are generally subclassified into macrocystic (cyst volume > 2 cm^2^), microcystic (cyst volume < 2 cm^2^), and mixed cystic (combination of both macrocysts and microcysts) [8,9]. cLMs are typically lined by a single or multiple layers of lymphatic endothelium. Macro cLM tends to be localized and usually causes simple compressive symptoms in adjacent tissues and organs [10,11]. By contrast, micro cLM demonstrates infiltrative characteristics, invading neighboring tissues with extensive involvement, ill-defined boundaries, and diffuse distribution patterns. It may induce abnormal proliferation of adjacent bone and soft tissues, posing greater therapeutic challenges [12,13,14]. Mixed cLM exhibits clinical features and progression patterns intermediate between macrocystic and microcystic types, with prognosis and treatment outcomes closer to those of macro cLM [15].

cLMs typically exhibit a relatively slow growth rate and rarely regress spontaneously [16]. However, when complicated by intracystic hemorrhage (ICH), it may rapidly enlarge in size over a short period, leading to acute compressive symptoms and associated pain [17,18,19]. ICH, as a specific manifestation of cLM, often occurs secondary to trauma or strenuous physical activity, while some cases present as spontaneous hemorrhage. Flocculent or punctate floating echoes and a mixed density/liquid–liquid level represent specific imaging features of ICH [20]. Characterized by an acute onset and rapid progression, its long-term impact on therapeutic outcomes in cLM currently lacks systematic investigation.

Due to the rarity of ICH in micro cLM, and its distinct treatment approaches compared to macro/mixed cLM [21,22,23], this study specifically investigated the impact of ICH on treatment efficacy and postoperative variables in pediatric cases with macro/mixed cLM. In addition, indocyanine green (ICG) lymphography was utilized to characterize lymph flow patterns in all cLM cases.

## 2. Patients and Methods

### 2.1. Patients

A retrospective cohort analysis was performed in all cases with macro/mixed cLM who underwent treatment in our center between January 2019 and January 2024. The clinical characteristics of enrolled cases were documented and included gender, age, histological typing, location, maximum diameter, and intracystic condition. Postoperative variables included duration of negative drainage, local infection, scar hyperplasia, and follow-up. All patients with/without ICH were diagnosed using preoperative imaging findings (ultrasonography [US], computed tomography [CT], or magnetic resonance imaging [MRI]) and uniformly received intraoperative ICG lymphography. The criteria for ICH determination included the following: (1) characteristic findings on preoperative imaging (flocculent or punctate floating echoes, liquid–liquid level) and (2) intraoperative identification of intracystic bloody lymph fluid. Both groups with/without ICH received consistent and standardized management (sclerotherapy combined with intracystic negative pressure), which induced continuous drainage to promote cyst shrinkage and avoid posttreatment accumulation of lymph [24].

The inclusion criteria included the following: (1) no previous intervention, (2) a macrocystic or mixed cystic type, (3) subcutaneous lesions, (4) intraoperative ICG lymphography, and (5) more than 6 months of post-treatment follow-up. The exclusion criteria included the following: (1) deep lesions (muscle involvement), (2) syndromic cLM, (3) history of iodine allergy, and (4) severe liver or kidney dysfunction. Our study was approved by the Ethics Committee of the Children’s Hospital of Nanjing Medical University (202503050-1), and was conducted in accordance with the precepts of the Helsinki Declaration. Written informed consent was obtained from the legal guardians of all patients.

### 2.2. ICG Fluorescence Imaging System

Under general anesthesia, 0.1 mL ICG solution (concentration: 2.5 mg/mL, Dandong Yichuang, China) was injected at multiple points distal to the cLM lesion by intradermal injection. The maximum dose of ICG was 0.5 mg/kg per session. A 10 min localized massage was administered in a distal-to-proximal direction, and ICG lymphography was performed using a near-infrared (NIR) fluorescence imaging system (Mingde Medical Diagnosis Inc., Langfang, China). During the massage, the skin was promptly wiped with alcohol-soaked gauze to avoid ICG artifact formation. ICG lymphography was commenced to assess the presence of lymph inflow or outflow connected to cLM lesions, with a real-time observation duration of approximately 10 min.

**Figure 1:**
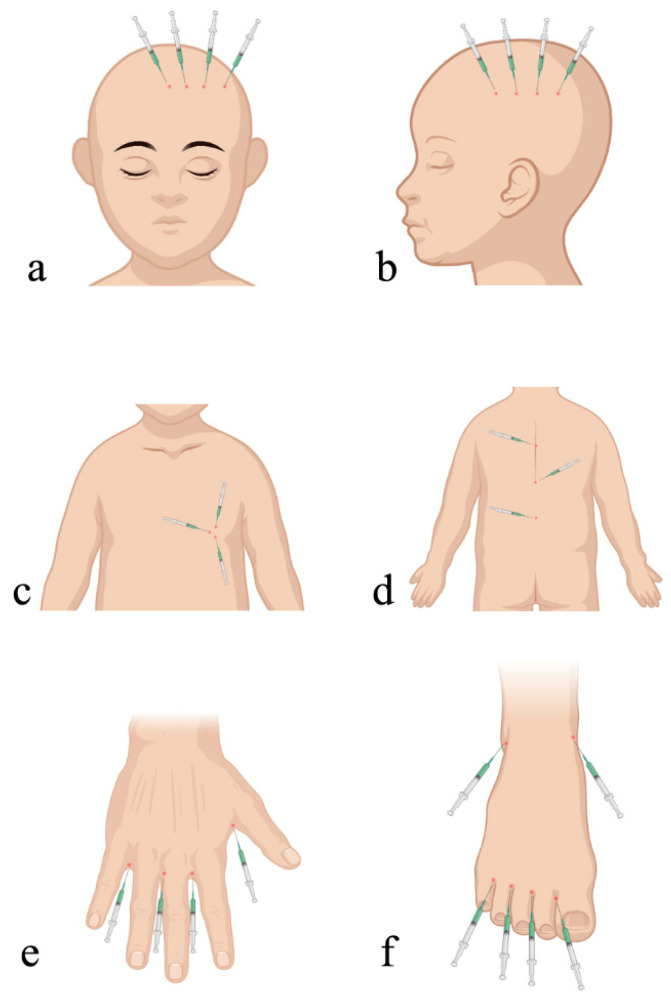
The selection of ICG injection points for cLM at the (**a**) face, (**b**) neck, (**c**) chest, (**d**) abdomen or back, (**e**) upper extremity, and (**f**) lower extremity. ICG, indocyanine green; cLM, cystic lymphatic malformation. Image drawn by Tao Han using BioRender.com.

The ICG injection points for cLM at specific anatomical regions were summarized as follows: (1) face: ipsilateral forehead scalp along the hairline; (2) neck: ipsilateral temporal scalp along the hairline; (3) chest: peri-areolar region on the ipsilateral side; (4) abdomen and back: multiple midline injection points distal to the lesion; (5) upper extremity: finger webs and antecubital fossa of the affected limb; and (6) lower extremity: toe webs, medial/lateral malleoli, and popliteal fossa of the affected limb (Figure 1).

### 2.3. Surgical Technique

Following ICG lymphography, a 1.5 to 2.0 cm linear incision was made over the surface of the cLM lesion. Through this incision, while protecting surrounding critical vessels and nerves, all cystic septa were penetrated under direct vision using electrocautery or scissors, followed by complete aspiration of intracystic lymphatic fluid. The cyst cavity was then cauterized twice with 5 to 20 mL 2% iodine tincture (20 mL; Guangdong South China Pharmacy Co, Ltd., Zhanjiang, China). After obtaining a small portion of cLM tissue for pathological examination, the wound was sutured, followed by the placement of an intracystic negative-pressure drainage tube.

### 2.4. Postoperative Management

On postoperative day 2, intracystic lavage with polidocanol (3%; Hameln Pharmaceutical Gmbh Co, Ltd., Wiesbaden, Germany) was performed via the drainage tube, followed by gentle massage of the lesion for 5 min to promote complete contact between the cavity and sclerosing agent. The polidocanol was then completely aspirated by reconnecting the drainage tube to negative pressure. This lavage procedure could be repeated every other day until minimal drainage output was observed. Between 7 and 10 days after the procedure, an injection of bleomycin (15 mg; Hisun Pfizer Co., Ltd., Hangzhou, China) was administered before the removal of the drainage tube. The amount of bleomycin that was injected was 0.5 to 1 mg/kg, and the maximum dose was limited to 15 mg per session. For all cases enrolled in this research study, the duration of postoperative follow-up was from 6 to 16 months.

### 2.5. Evaluation of Curative Effects

A physical examination was performed and US or MRI images were taken to carry out a postoperative evaluation of curative effects. An effective response was evaluated as follows: (1) excellent, a reduction in size of more than 90%; (2) good, a reduction in size ranging from 75 to 90%; (3) fair, a reduction in size ranging from 50 to 75%; and (4) poor, a reduction in size of <50% [25].

### 2.6. Statistical Analysis

The statistical analysis was performed with SPSS Statistics 22.0 (IBM Corp., Armonk, NY, USA). Categorical data, presented as n (%), were analyzed using a Chi-square test or Fisher’s exact test (expected counts were ≤5). The normality of the data was assessed using the Shapiro–Wilk test in this study, and continuous variables were expressed as mean ± standard deviation (SD). An independent two-sample *t*-test was performed for parametric comparisons. Univariate analysis was performed using a Chi-square test to evaluate the associations between gender, age, histological typing, lesion location, lesion maximum diameter, intracystic condition, and excellent outcome. With excellent outcome as the dependent variable, variables with a *p*-value < 0.05 in the univariate analysis were retained for multivariable binary logistic regression, and were presented using a forest plot. Differences were considered to be statistically significant at a *p*-value < 0.05.

## 3. Results

A total of 83 patients who met the inclusion criteria were analyzed during this study period (ICH group: *n* = 36 and without ICH group: *n* = 47). There were 54 (65.1%) males and 29 (34.9%) females, and the age of cases at diagnosis ranged from 1 to 151 months. Intravenous use of tranexamic acid (0.5 g; Sailong Co., Ltd., Yueyang, China) was utilized for preoperative hemostatic management (0.25 to 0.5 g/day, minimum 7-day course) in all cLM cases with ICH. The baseline characteristics of the patients are highlighted in Table 1. A comparison of demographic information and clinical characteristics between cases with and without ICH demonstrated that no significant difference was found between the two groups in terms of gender (*p* = 0.495), age (*p* = 0.157), histological typing (*p* = 0.820), lesion location (*p* = 0.972), and maximum diameter (*p* = 0.371), which indicated that the prognoses of these two groups were comparable.

**Figure 2:**
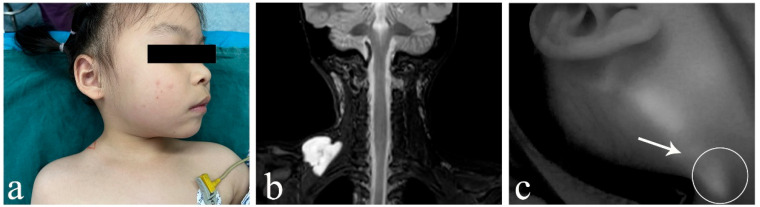

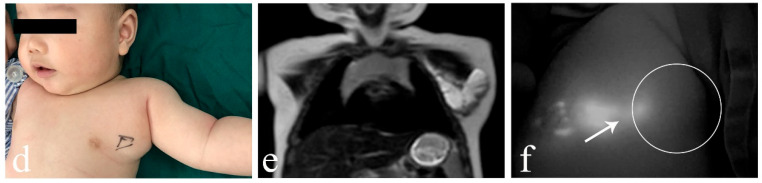
Images of macro/mixed cLM without ICH. (**a**) A 4-year-old female patient with macro cLM of the right neck. (**b**) Preoperative MRI indicated macro cysts without ICH. (**c**) Intraoperative ICG lymphography finding with one inflow from posterior auricular region draining into the lesion. (**d**) A 4-month-old female patient with mixed cLM of the left chest. (**e**) Preoperative MRI indicated mixed cysts without ICH. (**f**) Intraoperative ICG lymphography finding with one inflow from areola region draining into the lesion. *Arrow head*, lymph vessel; *circle*, region of the lesion. cLM, cystic lymphatic malformation; ICH, intracystic hemorrhage; MRI, magnetic resonance imaging; ICG, indocyanine green.

**Figure 3:**
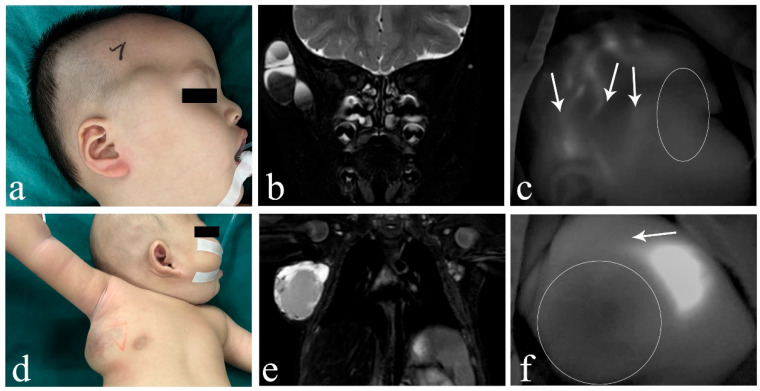
Images of macro/mixed cLM with ICH. (**a**) A 2-year-old male patient with macro cLM of the right temporal region. (**b**) Preoperative MRI indicated macro cysts with ICH. (**c**) Intraoperative ICG lymphography finding without any inflow draining into the lesion. (**d**) A 2-month-old male patient with mixed cLM of the right chest. (**e**) Preoperative MRI indicated mixed cysts with ICH. (**f**) Intraoperative ICG lymphography finding without any inflow draining into the lesion. *Arrow head*, lymph vessel; *circle*, region of the lesion. cLM, cystic lymphatic malformation; ICH, intracystic hemorrhage; MRI, magnetic resonance imaging; ICG, indocyanine green.

In cLM cases without ICH, ICG lymphography consistently identified at least one lymphatic inflow draining into the lesion (five cases with inflows ≥ 2), regardless of whether the histological typing was macrocystic or mixed cystic. Both the morphological characteristics and fluorescence intensity of the lymphatic inflows appeared within normal limits, showing no significant difference from nearby superficial lymphatic vessels (Figure 2). However, in ICH cases (both macrocystic and mixed cystic), a complete absence of afferent lymphatic vessels was demonstrated using ICG lymphography, suggesting a lack lymphatic system communication and an isolated nature (Figure 3). It is noteworthy that no lymphatic outflow was detected in any of the cases (with/without ICH) involved in this study.

**Figure 4:**
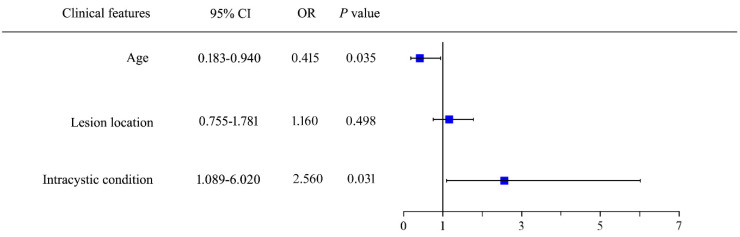
Forest plot from multivariable binary logistic regression analysis in cLM cases with excellent outcomes. OR, odds ratio; CI, confidence interval.

**Figure 5:**
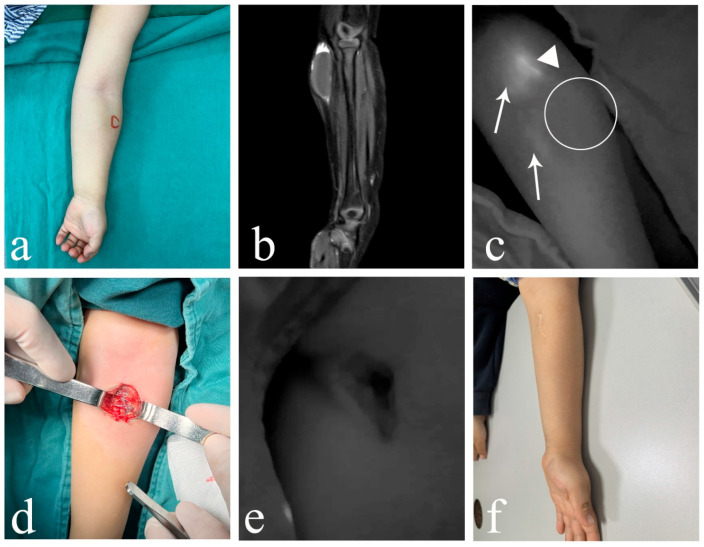
A 2-year-old male patient suffered from macro cLM with ICH in left forearm. (**a**) Clinical appearance preoperatively. (**b**) Preoperative MRI indicated macro cysts with ICH. (**c**) ICG lymphography showing that one lymph vessel on the ulnar side of the left forearm bypassed the lesion and flowed into the cubital fossa lymph node. No communication between lymph vessel and the lesion cite was observed. (**d**) Gross observation of ICH intraoperatively. (**e**) No ICG was found inside the lesion. (**f**) Twelve months postoperatively, complete regression of cLM met the criterion of excellent curative effect. *Arrow head*, lymph vessel; *circle*, region of the lesion; *triangle*, lymph node. cLM, cystic lymphatic malformation; ICH, intracystic hemorrhage; MRI, magnetic resonance imaging; ICG, indocyanine green.

**Figure 6:**
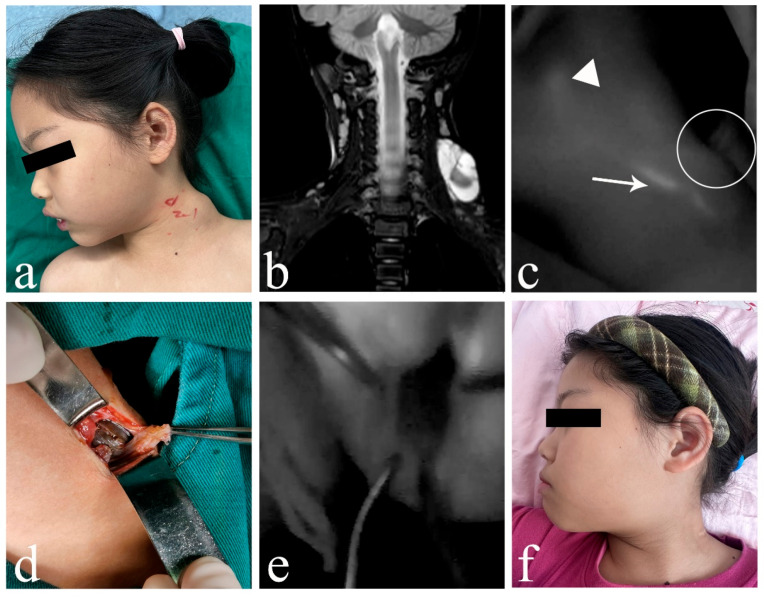
A 7-year-old female patient suffered from mixed cLM with ICH in left neck. (**a**) Clinical appearance preoperatively. (**b**) Preoperative MRI indicated mixed cysts with ICH. (**c**) ICG lymphography showed that one lymph vessel from posterior auricular region bypassed the lesion, and no communication between lymph vessel and the lesion cite was observed. (**d**) Gross observation of ICH intraoperatively. (**e**) No ICG was found inside the lesion. (**f**) Sixteen months postoperatively, complete regression of cLM met the criterion of excellent curative effect. *Arrow head*, lymph vessel; *circle*, region of the lesion; *triangle*, lymph node. cLM, cystic lymphatic malformation; ICH, intracystic hemorrhage; MRI, magnetic resonance imaging; ICG, indocyanine green.

**Figure 7:**
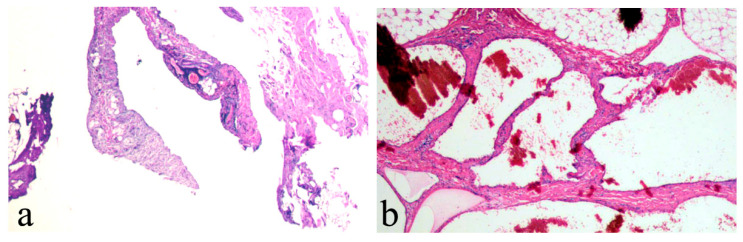
Postoperative pathology. (**a**) cLM without ICH: structure of lymphatic vessel wall and lymphoid tissue. (**b**) cLM with ICH: similar lymphatic vessel wall structure and lymphoid tissue, accompanied by intracystic blood clots. cLM, cystic lymphatic malformation; ICH, intracystic hemorrhage.

To understand the clinical characteristics for curative effects in cLM cases, a univariate analysis (Chi-square t-tests) was conducted. For preoperative predictors of the excellent outcome variable, Table 2 revealed significant differences in age (*p* = 0.017), lesion location (*p* = 0.026), and intracystic condition (*p* = 0.020), while no significant variations were observed for gender (*p* = 1.000), histological typing (*p* = 0.246), or maximum diameter (*p* = 0.176). Moreover, compared to the group without ICH, the ICH group required significantly fewer subsequent treatment sessions (5.6% versus 27.7%, *p* = 0.010). As shown in Figure 4, we conducted a multivariable binary logistic regression analysis of three clinical factors and identified two independent preoperative predictors of excellent outcome: young age (*p* = 035; OR, 0.415; 95% CI, 0.183–0.940) and ICH (*p* = 031; OR, 2.560; 95% CI, 1.089–6.020). Hence, ICH was identified as the main predictor, and younger patients (0–3 years old) had a lower potential for postoperative excellent outcomes (Figure 5 and Figure 6). Postoperative pathological examinations were performed in all enrolled cases, with histological parameters (similar lymphatic vessel wall structure and lymphoid tissue between two groups) matching normal cLM lesions. The sole distinguishing feature was the presence of intracystic scattered blood clots in cLM with ICH (Figure 7).

In the comparison of postoperative variables (Table 3), the ICH group exhibited a significant reduction in duration of negative drainage (5.42 ± 1.36 versus 6.98 ± 2.17, *p* < 0.001). However, no significant intergroup differences were observed in complication profiles, including local infection (*p* = 0.693) and scar hypertrophy (*p* = 0.648). Temporary ICG-related cutaneous hyperpigmentation was observed in all cases, with spontaneous resolution within 30 days after the procedure. No adverse events met the criteria for ICG allergy or hepatorenal impairment.

## 4. Discussion

The primary therapeutic objectives for cLM are to provide symptomatic relief, achieve regression of the majority of the lesions, and thereby restore local appearance and function [21,26]. Current therapeutic approaches primarily include surgical resection, sclerotherapy (e.g., bleomycin, doxycycline, OK-432, 2% iodine tincture), radiofrequency ablation, laser therapy, and topical/oral sirolimus [24,27,28,29,30,31]. However, cLMs located in cervicofacial or oropharyngeal regions pose unique challenges due to their deep-seated location, extensive infiltration, and proximity to vital neurovascular structures. The ill-defined margins and invasive growth pattern of these lesions often render postoperative excellent outcomes unachievable with standalone surgical or sclerotherapy interventions. Often a combined treatment is needed. Notably, repeated treatments are needed in many cases, yet postoperative lymph re-accumulation and local recurrence support the notion that cLM retains functional connections with the native lymphatic drainage system [32,33].

The primary imaging techniques for the diagnosis of cLM include US, CT, and MRI. These modalities primarily characterize cystic cavities, internal septations, and intracystic conditions. MRI is particularly valuable for preoperative assessments, providing detailed information on cLM histological typing and anatomical relationships with adjacent tissues [34,35]. Notably, on contrast-enhanced scans, mild enhancement can always be observed in the cyst wall or separations, suggesting the presence of vascular distribution within the wall, which may serve as a potential source of ICH [36,37].

cLM with ICH is more prone to rapid increases in size, resulting in acute pain and local compression symptoms, which have been reported frequently in previous studies [38,39]. However, the impact of ICH on the long-term prognosis of cLM lacks systematic evaluation. As observed in our comparative study, the ICH group was more prone to better postoperative outcomes, fewer subsequent treatments, and shorter durations of negative drainage. Our results indicate that ICH was a contributing factor for excellent outcomes, especially in macro or mixed cLM cases. The differences in the therapeutic outcomes can be seen in our univariate and multivariate analyses between the two groups with/without ICH.

This observation demonstrated that the severity of ICH may be greatly overestimated owing to its obvious symptoms and rapid progression. However, the mechanisms underlying its potential positive impact on the therapeutic efficacy against cLM remain unclear and warrant further investigation. Kato et al. classified the lymphatic flow patterns based on ICG lymphography findings in 20 pediatric patients with cLM. In his research, type 1 (single inflow) and type 2 (multiple inflows) cLMs were connected to lymphatic system, whereas inflow was not detectable in types 3 or 4 [40]. In the present study, we performed ICG lymphography to investigate the lymph flow patterns of all cLM cases. Our findings revealed that the isolated nature of ICH cases appeared to naturally disrupt connections with the lymphatic drainage system, potentially accounting for the ICH group’s favorable therapeutic outcomes, which were characterized by reduced postoperative lymph re-accumulation and lower recurrence rates. Moreover, the potential role of hemorrhage in promoting cystic fibrosis, a non-negligible factor that may facilitate cyst shrinkage, warrants careful consideration. Postoperative pathological comparisons revealed no structural alterations but showed scattered blood clots in cLM with ICH. We hypothesized that intracystic blood clots and thrombi may obstruct the afferent lymphatic vessels, potentially accounting for ICH lesion isolation. However, whether isolated cLM lesions are congenital or secondary to ICH requires further validation through our subsequent animal experiments. Furthermore, our findings raise the possibility that selective occlusion of afferent lymphatic vessels may potentiate existing treatment modalities for cLM cases without ICH, warranting systematic evaluation in future studies.

Several limitations of this research deserve comment. First, this single-center research study may limit the generalizability of our results to other centers. Second, the retrospective nature made this research study susceptible to reporting bias. Third, the relatively short follow-up period may have restricted the ability to assess long-term outcomes or the delayed effects of cLM management. In addition, a limiting property of ICG fluorescence (limited to 10 mm for visualization) precluded its application in assessing lymphatic pathways of deep cLM, necessitating the exclusion of intramuscular and intra-thoracoabdominal cases from our cohorts. Furthermore, micro cLM cases with ICH were excluded given their rarity (*n*= 4; 1 vaginal/3 lingual) and differing treatment modalities, so the results cannot be generalized to all kinds of histological typing for cLM.

## 5. Conclusions

ICH emerges as a favorable independent prognostic predictor in macro/mixed cLM management. Although characterized by aggressive progression and compressive symptoms, ICH exhibits superior long-term therapeutic outcomes following treatment, potentially attributable to its isolated nature.

## Figures and Tables

**Table 1:** Baseline clinical characteristics of the research subjects.

Characteristics	ICH (*n* = 36)	Without ICH (*n* = 47)	X^2^	*p* Value
Gender			0.538	0.495
Male	25	29		
Female	11	18		
Age (years)			2.418	0.157
0~3	21	35		
>3	15	12		
Histological typing			0.072	0.820
Macrocystic	24	30		
Mixed cystic	12	17		
Lesion location			0.057	0.972
Cervicofacial region	20	25		
Extremities	7	10		
Trunk	9	12		
Maximum diameter (cm)			1.030	0.371
≤5	17	17		
>5	19	30		

*ICH* intracystic hemorrhage.

**Table 2:** Univariate analysis of clinical characteristics for curative effects in cLM cases.

Characteristics	*n*	Excellent Outcome (*n*, %)	Subsequent Treatment (*n*, %)
Gender			
Male	54	31 (57.4)	9 (16.7)
Female	29	17 (58.6)	6 (20.7)
Age (years)			
0~3	56	* 27 (48.2)	12 (21.4)
>3	27	21 (77.8)	3 (11.1)
Histological typing			
Macrocystic	54	34 (63.0)	8 (14.8)
Mixed cystic	29	14 (48.3)	7 (24.1)
Lesion location			
Cervicofacial region	45	* 20 (44.4)	8 (17.8)
Extremities	17	13 (76.5)	2 (11.8)
Trunk	21	15 (71.4)	5 (23.8)
Maximum diameter (cm)			
≤5	34	23 (67.6)	4 (11.8)
>5	49	25 (51.0)	11 (22.4)
Intracystic condition			
ICH	36	* 26 (72.2)	* 2 (5.6)
Without ICH	47	22 (46.8)	13 (27.7)

*ICH*, intracystic hemorrhage. Values are given in numbers (proportion). * Indicates *p* < 0.05.

**Table 3:** Comparison of postoperative variables between the two groups.

Characteristics	ICH (*n* = 36)	Without ICH (*n* = 47)	*p* Value
Duration of negative drainage (days)	5.42 ± 1.36	6.98 ± 2.17	<0.001
Local infection (*n*)	2 (5.6)	4 (8.5)	0.693
Scar hyperplasia (*n*)	3 (8.3)	2 (4.3)	0.648
Follow-up (months)	9.00 ± 2.69	9.49 ± 3.05	0.449

*ICH* intracystic hemorrhage. Values are given in numbers (proportion) or mean (standard deviation).

## Data Availability

The original contributions presented in this study are included in the article. Further inquiries can be directed to the corresponding author.
